# RETRACTION: The Dynamics of Type 2 Diabetes Mellitus Prevalence and Management Rates among Rural Population in Henan Province, China

**DOI:** 10.1155/jdr/9804834

**Published:** 2026-08-20

**Authors:** Journal of Diabetes Research

RETRACTION: X. Liu, L. Wang, P. Wang, R. Liu, K. Yang, X. Qian, J. Fan, S. Yu, Y. Li, C. Wang, “The Dynamics of Type 2 Diabetes Mellitus Prevalence and Management Rates among Rural Population in Henan Province, China,” *Journal of Diabetes Research*, 2017, 9092759, https://doi.org/10.1155/2017/9092759

The above article, published online on 23 February 2017 in Wiley Online Library (wileyonlinelibrary.com), has been retracted by agreement between the journal Associate Editor, Andrea Scaramuzza; and John Wiley & Sons Ltd. The retraction was intitially requested by the authors due to not owning the data used in the study, and not obtaining permission from the data owners to publish the reported results. The investigation into these concerns was subsequently paused for a number of years. The authors have not responded to recent correspondence regarding the outcome our investigation and the decision to retract the article.

